# Unveiling *CKS2* : A Key Player in Aggressive B‐Cell Lymphoma Progression and a Target for Synergistic Therapy

**DOI:** 10.1002/cam4.70435

**Published:** 2024-11-19

**Authors:** Fenling Zhou, Lu Chen, Zhen Liu, Yuli Cao, Cuilan Deng, Gexiu Liu, Chengcheng Liu

**Affiliations:** ^1^ Department of Hematology Sun Yat‐Sen Institute of Hematology, The Third Affiliated Hospital of Sun Yat‐Sen University Guangzhou Guangdong People's Republic of China; ^2^ Institute of Hematology Jinan University Guangzhou Guangdong People's Republic of China; ^3^ Department of Hematology, First Affiliated Hospital Jinan University Guangzhou Guangdong People's Republic of China

**Keywords:** bioinformatics analysis, Burkitt cell lymphoma (BL), cyclin‐dependent kinases regulatory subunit 2 (*CKS2*), diffuse large B‐cell lymphoma (DLBCL), etoposide, targeted therapy

## Abstract

**Background:**

The objective of this study was to investigate the expression levels and biological significance of *CKS2* in Burkitt cell lymphoma (BL) and diffuse large B‐cell lymphoma (DLBCL). Additionally, the potential synergistic anti‐tumor effects of *CKS2* knockdown in combination with etoposide in BL and DLBCL were explored for the first time.

**Methods:**

Bioinformatics analysis was utilized to explore the transcriptional levels, prognostic value, and gene function enrichment of *CKS2* in BL and DLBCL. Specific shRNA sequences were designed to target *CKS2* for the purpose of constructing a lentiviral expression vector, and therapeutic effects were assessed through analyses of cell proliferation, cell cycle distribution, and cell apoptosis.

**Results:**

First, the study examined the increased transcriptional and protein levels of *CKS2* in BL and DLBCL through analysis of various databases and immunohistochemistry tests. Elevated *CKS2* expression was found to be correlated with a worse prognosis in BL and DLBCL patients, as evidenced by data from the TCGA and GEO databases. Enrichment analysis indicated that *CKS2* functions were primarily linked to protein kinase regulatory activity, G1/S phase transition of the cell cycle, and the p53 signaling pathway, among others. Second, stable suppression of *CKS2* gene expression in Raji and SUDHL6 cells using shRNA resulted in a significant inhibition of cell proliferation. Moreover, *CKS2*‐shRNA induced G0/G1 cell cycle arrest and apoptosis by activating the p53 signaling pathway in Raji and SUDHL6 cells. Third, the combined treatment of *CKS2*‐shRNA and etoposide exhibited a synergistic effect on the proliferation and apoptosis of Raji and SUDHL6 cells.

**Conclusions:**

Our findings suggest that *CKS2* may play a critical role in the progression of BL and DLBCL and provide evidence for the potential therapeutic application of combining *CKS2*‐shRNA and etoposide agents in the treatment of BL and DLBCL.

**Abbreviations**
AOD: average optical density
BL: Burkitt cell lymphoma
BP: biological process
CC: cellular component
CCLE: Cancer Cell Line Encyclopedia
CDKs: cyclin‐dependent kinases
*CKS2*
: cyclin‐dependent kinases regulatory subunit 2
DAB: diaminobenzidine
DLBCL: diffuse large B‐cell lymphoma
Dm: dose of median
FDR: false discovery rate
GAPDH: glyceraldehyde 3‐phosphate dehydrogenase
GEO: gene expression omnibus
GEPIA: gene expression profiling interactive analysis
GO: Gene Ontology
HR: hazard ratio
IC50: 50% inhibition concentration
IHC: immunohistochemical
KEGG: Kyoto Encyclopedia Gene and Genome
MF: molecular function
NHL: Non‐Hodgkin's lymphoma
OS: overall survival
PI: propidium iodide
PPIs: protein–protein interactions
RNAi: RNA interference
shRNA: lentivirus‐delivered short hairpin RNA

## 1 Background

Non‐Hodgkin's lymphoma (NHL), an assortment of heterogeneous malignancies derived from lymphoid tissue, ranks as the seventh most common form of cancer [1]. In terms of origin, NHL can be categorized into three types: B‐cell origin, T‐cell origin, and natural killer‐cell origin [2]. Among these cases, around 80%–90% originate from B‐cells, specifically termed B‐NHL [3, 4]. Notably, the most prevalent forms of B‐NHL include Burkitt cell lymphoma (BL), diffuse large B‐cell lymphoma (DLBCL), follicular lymphoma, chronic lymphocytic leukemia/small lymphocytic lymphoma, and mantle lymphoma [2]. Based on incidence trends, approximately 65,000 new B‐NHL patients are affected each year in the United States alone [1]. Despite numerous trials in treating B‐NHL, the comprehensive understanding of its diagnosis, prognosis, and underlying mechanism remains incomplete due to its varied clinical and pathological presentations. Chemoresistance is a leading cause of treatment failure in B‐NHL, to the extent that even one of the primary chemotherapeutic agents for these cancers, etoposide, has only marginal effects on clinical prognosis [5, 6].

Etoposide, a commonly prescribed anticancer medication that targets type II topoisomerases, continues to be the primary choice for first‐line treatment in small‐cell lung cancer [7]. In recent years, mounting evidence has substantiated the beneficial impact of etoposide in the treatment of B‐cell lymphoblastic leukemia, T‐cell lymphoma, chronic myeloid leukemia, and other conditions [8, 9, 10, 11]. This results from its ability to impair DNA damage repair and decrease the proliferation of malignant cells. Cyclin‐dependent kinase regulatory subunit 2 (*CKS2*), located in chromosome 9q22, is a highly conserved human cyclin‐dependent kinase‐binding protein family member [12]. *CKS2* may serve an essential role in somatic cell division, meiosis, and early embryonic development [13]. Indeed, numerous studies have reported abnormal overexpression of *CKS2* in various malignant tumors, where it has been implicated in promoting tumor progression and drug resistance [14, 15]. For example, recent research by Jonsson et al. has unveiled an intriguing link between the regulation of cell division by nuclear pathways and oxidative phosphorylation in the mitochondrion that involves *CKS2* and promotes chemoradioresistance of cervical cancer [16]. Compared to normal human lymphoid cells, malignant lymphoid cells exhibit the elevated *CKS2* expression levels, which are associated with increased cell proliferation. However, the precise underlying mechanism driving this phenomenon remains incompletely understood [13]. Against this backdrop, our study seeks to explore, for the first time, the expression levels and biological significance of CKS2 in BL and DLBCL. Concurrently, we investigate whether direct targeting of *CKS2* can enhance the sensitivity of BL and DLBCL to etoposide, potentially enhancing their chemotherapy response.

Lentivirus‐delivered short hairpin RNA (shRNA) has emerged as a highly specific post‐transcriptional gene expression suppression strategy [17, 18]. Further, the feasibility of reversing chemoresistance using shRNA‐based strategies has been demonstrated in vitro [19]. In our study, we found that shRNA‐mediated *CKS2* silencing could inhibit proliferation and induce G0/G1 phase arrest and cell apoptosis in BL and DLBCL cells by activating the p53 signaling pathway. Furthermore, we sought to determine whether *CKS2*‐specific shRNA‐based treatment would be an effective strategy to enhance the sensitivity of BL and DLBCL to etoposide. Our study reveals, for the first time, that shRNA targeting *CKS2* increased etoposide‐induced cytotoxicity in vitro. These findings underscore *CKS2* as a promising therapeutic target in BL and DLBCL, highlighting shRNA‐mediated gene silencing as a potent anti‐tumor strategy in experimental settings.

## 2 Materials and Methods

### 2.1 Bioinformatics Analysis

#### 2.1.1 Gene Expression Analysis Including Raji and SUDHL6 Cells

Gene Expression Profiling Interactive Analysis (GEPIA, www.gepia.cancer‐pku.cn) is a recently developed interactive web server that encompasses mRNA expression data from 9736 tumors and 8587 normal samples sourced from the Cancer Genome Atlas and the Genotype Tissue Expression projects [20]. In September 2021, we employed the ‘Expression on Box Plots’ module to examine the mRNA expression of the *CKS2* gene across various common tumors. A *p*‐value of < 0.05 was deemed statistically significant.

The Cancer Cell Line Encyclopedia (CCLE) database includes RNA‐seq data from 1457 cancer cell lines [21]. In this study, we investigated the mRNA expression levels of *CKS2* across diverse cancer cell lines, including Raji and DLBCL, in May 2021.

In September 2021, we used the Gene Expression Omnibus (GEO) database to compare the mRNA expression levels of *CKS2* in tumor tissues with those in normal control: GSE9327 (9 BL samples, 36 DLBCL samples, and 8 normal lymph nodes) [22]; GSE12453 (5 BL samples, 11 DLBCL samples, 5 naive B‐cells, 5 memory B cells, and 5 plasma cells) [23]; GSE56315 (55 DLBCL samples and 7 memory B cells) [24]; GSE44337(9 DLBCL samples and 3 Memory B‐cells); GSE32018 (22 DLBCL samples and 7 Lymph‐node) [25].

In September 2021, we employed EMBL‐EBI (https://www.ebi.ac.uk) for an in‐depth analysis of *CKS2* gene expression in eight commonly studied Raji and DLBCL cell lines. The extensive collection of cancer cell lines in this database offers valuable insights into gene expression across different cancer subtypes of various tissue origins [26].

#### 2.1.2 Survival Prognosis Analysis

In October 2021, data from The Cancer Genome Atlas (TCGA, http://cancergenome.nih.gov/) were obtained to integrate the expression of *CKS2* with BL patient survival data. Additionally, data from the GEO (GSE10846) were acquired to integrate the expression of *CKS2* with DLBCL patient survival data [27]. Survival analysis was performed with the survival R package and using Kaplan–Meier analysis (log‐rank test).

#### 2.1.3 CKS2‐Related Gene Enrichment Analysis

Data regarding CKS2 protein–protein interactions (PPIs) were retrieved from the GeneMANIA database in October 2024 [28]. Gene Ontology (GO) terms and Kyoto Encyclopedia of Genes and Genomes (KEGG) pathway enrichment analysis were conducted on CKS2 and its 50 neighboring genes using the DAVID 6.8 tool in October 2021 [29]. GO describes the genes in three ways, namely biological process (BP), molecular function (MF), and cellular component (CC). Significance was determined by a false discovery rate (FDR) of < 0.05.

### 2.2 Experimental Verification

#### 2.2.1 Immunohistochemistry

The clinical samples (15 non‐tumor lymph nodes, 10 paraffin BL tissues, and 32 paraffin DLBCL tissues) were collected from the Department of Pathology, The First Affiliated Hospital of Jinan University. All patients provided informed consent for the study, which got the approval of the Research Ethics Committee of Jinan University. The tissue sections were paraffinized and soaked in 1× EDTA antigen retrieval solution for 20 min to retrieve the cell antigens. After blocking, the antigen–antibody reaction was incubated overnight at 4°C. Immunohistochemical staining of tissue sections was performed using 3,3′‐diaminobenzidine (DAB) solution, and all sections were counterstained with hematoxylin. Rabbit monoclonal antibodies against CKS2 (ab155078, Abcam) were utilized at a dilution of 1:50. At higher magnification (400×), ten randomly selected visual fields were analyzed for positive signal expression using ImageJ software. Protein expression in non‐tumor lymph node tissues, BL tissues and DLBCL tissues was compared based on the average optical density (AOD) as a parameter for semi‐quantitative detection.

#### 2.2.2 Cell Lines and Cell Culture

The human BL cell line Raji and DLBCL cell line SUDHL6 were obtained from the Institute of Hematology, Jinan University (Guang Zhou, China). These cells were cultured in RPMI‐1640 medium (Gibco, USA) under 5% carbon dioxide at 37°C, supplemented with 10% fetal bovine serum (Gibco, USA). The culture medium was refreshed every 24 h, and cell passaging was performed every 2–3 days.

#### 2.2.3 Transfection and Stable Cell Lines

*CKS2*‐shRNA1, *CKS2*‐shRNA2, and scrambled control‐shRNA (NC‐shRNA) were purchased from Shanghai Jikai Company. *CKS2*‐shRNA1, *CKS2*‐shRNA2, and NC‐shRNA were designed and synthesized as follows: *CKS2*‐shRNA1, 5′‐GCTCAGTTAAATGCAACTGCA‐3′ and antisense, 5′‐TGCAGTTGCATTTAACTGAGC‐3′; *CKS2*‐shRNA2, 5′‐GAGTCTAGGCTGGGTTCATTA‐3′ and antisense, 5′‐GTAATGAACCCAGCCTAGACTC‐3′; NC‐shRNA sense, 5′‐CCTAAGGTTAAGTCGCCCTCGC‐3′, and antisense, 5′‐GCGAGGGCGACTTAACCTTAGG‐3′. For shRNA transfection, 293 T cells were cultured to 80% confluence in a 10 cm petri dish and transfected with NC‐shRNA, *CKS2*‐shRNA1, and *CKS2*‐shRNA2 using Lipofectamine 2000 Reagent (Invitrogen; Thermo Fisher Scientific Inc.) for 48 h. Subsequently, the lentivirus supernatant was collected, concentrated, and added to Raji and SUDHL6 cells for 72 h. Following this, the cells were subjected to selection with 5 μg/mL puromycin for 1 month, until green fluorescence expression could be observed in all cells under a fluorescence microscope. Subsequent experiments could then be conducted.

#### 2.2.4 qRT‐PCR Analysis

Total RNA was extracted from using a total RNA extraction kit, following the manufacturer's instructions (TRIzol). The absorbance at 260 and 280 nm was measured to determine RNA concentration. Reverse transcription was carried out to generate cDNA, as per the instructions provided with the reverse transcription kit. Primer sequences for *CKS2* and glyceraldehyde 3‐phosphate dehydrogenase (GAPDH) were designed and synthesized by Tsingke Biotechnology Co. Ltd. (Beijing, China). The primer sequences for *CKS2* were as follows: forward, 5′‐TTCGACGAACACTACGAGTACC‐3′; reverse, 5′‐GGACACCAAGTCTCCTCCAC‐3′. Primer sequences for GAPDH were as follows: forward, 5′‐GGAGCGAGATCCCTCCAAAAT‐3′; reverse, 5′‐GGCTGTTGTCATACTTCTCATGG‐3′. Primer sequences for p53 were as follows: forward, 5′‐GCCCATCCTCACCATCATCACAC; reverse, 5′‐GCACAAACACGCACCTCAAAGC. The PCR reaction system was prepared following the instructions provided in the SYBR Premix Ex Taq (Accurate Biology, China) kit. Relative expression modifications were estimated employing the 2^−ΔΔCt^ method.

#### 2.2.5 Western Blot Assay

Raji and SUDHL6 cell samples were lysed on ice for 30 min using RIPA lysis buffer (Beyotime Biotechnology, Shanghai, China). Then, lysed cells were centrifugated at 13,000×*g* at 4°C for 10 min. Once the supernatant was obtained, a BCA assay kit (KeyGEN Biotechnology, Jiangsu, China) was utilized to measure the protein concentration. Proteins were then transferred onto PVDF membranes (Merck, China). The membrane was incubated with primary antibodies (1:1000) and secondary antibodies (1:2000) at 4°C overnight. Immunoreactive bands were detected by enhanced chemiluminescence (ECL) (Thermo Fisher Scientific, China) and photographed with an image acquisition system. Antibodies against proteins are listed in Table S1.

#### 2.2.6 Determination of Dose of Median (Dm) of Etoposide

Cells were seeded into 96‐well plates at a density of approximately 5 × 10^4^ cells/mL. Etoposide was added at final concentrations of 2, 4, 6 and 8 μmol/L. Untreated cells were included as a control. Forty‐eight hours later, 10 μL CCK8 was added into each well and incubated for 4 h at 37°C. The absorbance at 450 nm was determined using a Microplate Reader (Bio‐Rad 680). Then, 50% inhibition concentration (IC50) of etoposide, as referred to Dm, was calculated. Cell‐line experiments were triplicated.

#### 2.2.7 Cell Counting Kit‐8 Assay

Cellular proliferation was assessed using the CCK‐8 assay following the manufacturer's instructions (Beyotime Biotechnology, China). Raji and SUDHL6 cell were added to each well of a 96‐well plate and incubated for 24 h, followed by the addition of 10 μL of the kit reagent, which was incubated in the dark for 1 h. The average optical density (AOD) was measured at 450 nm. The CCK‐8 kit was employed to evaluate the proliferation of Raji and SUDHL6 cells at 24, 48, and 72 h after shRNA transfection.

#### 2.2.8 Cell Cycle Analysis and Apoptosis Analysis

For cell cycle analyses, Raji and SUDHL6 cells were seeded in 6‐well plates. After incubation, the cells were washed with PBS and centrifuged at 500×*g* for 5 min at 4°C. Then, the 100 μL DNA staining solution and 1 μL permeabilization were re‐introduced for 30 min in the dark. The cell cycle was measured by flow cytometric analysis of the DNA content of cell populations. Finally, the distribution of cells within G1/S and G2 and M phases was determined by using Flowjo software.

For apoptosis analysis, Raji and SUDHL6 cells were cultured in 6‐well culture plates. After incubation, the cells were washed with PBS and centrifuged at 500×*g* for 5 min at 4°C. Then, the cell suspension was re‐suspended with 1× binding buffer, followed by the addition of 10 μL Annexin‐V‐APC and 20 μL propidium iodide (PI) (Multi science, China) for 5 min in the dark. The apoptotic cells were quantified using flow cytometry.

### 2.3 Statistical Analysis

GraphPad Prism software version 8.0 (GraphPad Software Inc. San Diego, CA, USA) was used for statistical analysis and visualization. Statistical analysis between two samples was performed using the Student's *t*‐test. Statistical comparison of more than two groups was performed using one‐way ANOVA. Overall survival rate was calculated according to the Kaplan–Meier method and the difference in survival curves was evaluated by the log‐rank test. *p* < 0.05 was considered to indicate statistically significant differences.

## 3 Results

### 3.1 *CKS2* mRNA Expression Was Significantly Up‐Regulated in BL and DLBCL

**FIGURE 1:**
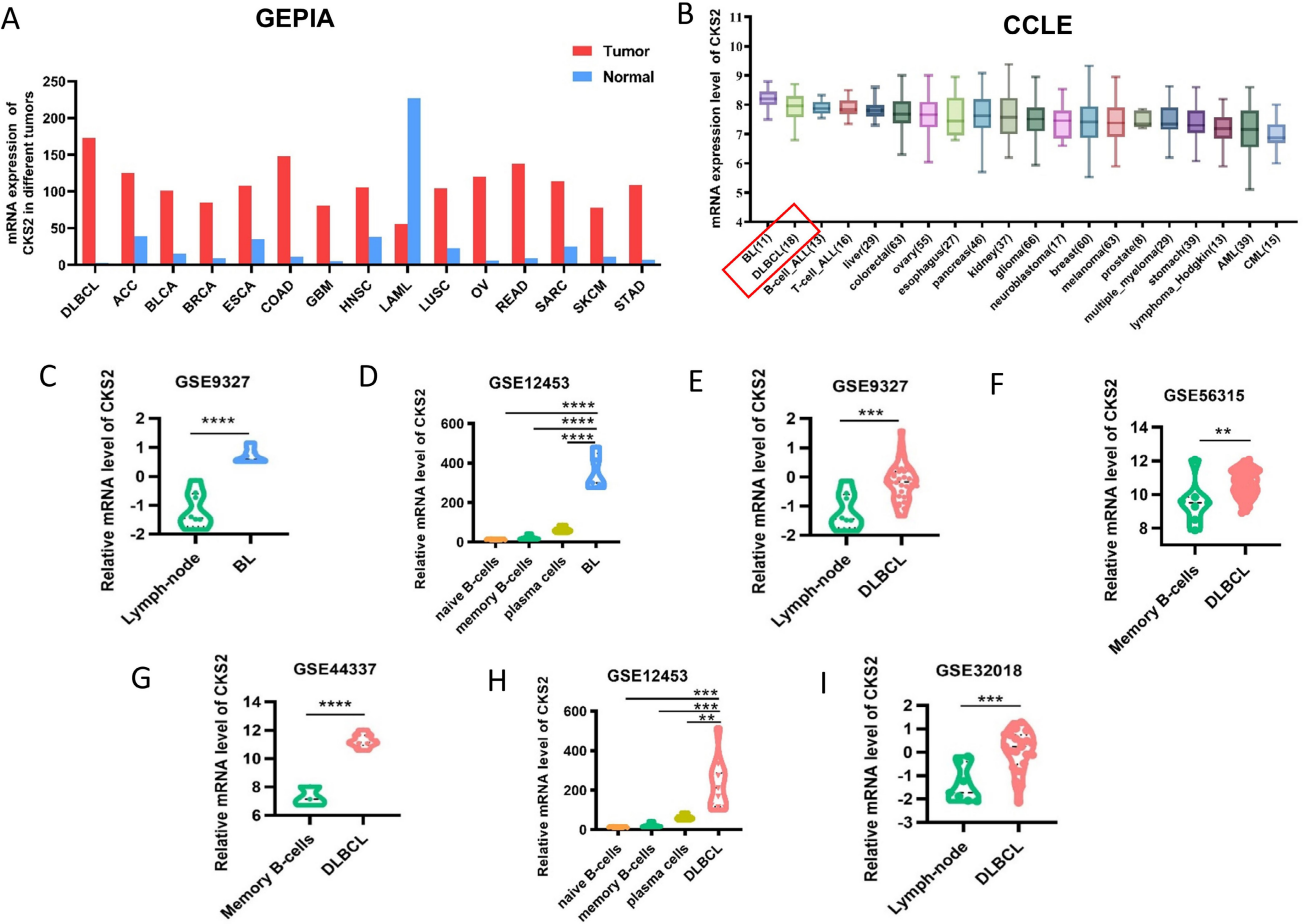
Investigation of *CKS2* gene expression in tumors using bioinformatics. (A) Analysis of *CKS2* gene expression in diverse tumor types. (B) *CKS2* gene expression in a range of tumor cell lines, including BL and DLBCL. (C–I) Elevated *CKS2* gene expression in BL and DLBCL based on GEO database data. *Statistical significance denoted as follows: **p < 0.01, ***p < 0.001, ****p < 0.0001. ACC, adrenocortical carcinoma; ALL, acute lymphoblastic leukemia; AML, acute myeloid leukemia; BL, Burkitt cell lymphoma; BLCA, bladder urothelial carcinoma; BRCA, breast invasive carcinoma; CML, chronic myeloid leukemia; COAD, colon adenocarcinoma; DLBCL, diffuse large B‐cell lymphoma; ESCA, esophageal carcinoma; GBM, glioblastoma multiforme; HNSC, head and neck squamous cell carcinoma; LAML, acute myeloid leukemia; LUSC, lung squamous cell carcinoma; OV, ovarian serous cystadenocarcinoma; READ, rectum adenocarcinoma; SARC, sarcoma; SKCM, skin cutaneous melanoma; STAD, stomach adenocarcinoma.

The GEPIA2 database revealed a notable upregulation of the *CKS2* gene across various tumors (Figure 1A). However, there has been limited documentation regarding the expression of the *CKS2* gene in B‐cell lymphomas. Through an analysis of the CCLE database, we discovered high expression levels of *CKS2* in B‐cell lymphomas, with the BL cell line ranking first and the DLBCL cell line ranking second (Figure 1B).

Subsequently, we conducted a detailed examination of *CKS2* expression in BL and DLBCL. As depicted in Figure 1C–I, the mRNA expression levels of *CKS2* in BL tissues were significantly elevated compared to those in normal lymph nodes or lymphocytes, as observed in GSE9327 and GSE12453 datasets. Moreover, the analysis demonstrated a consistently high mRNA expression of *CKS2* in DLBCL across multiple GEO datasets (GSE9327, GSE56315, GSE44337, GSE12453, GSE32018).

### 3.2 *CKS2* Expression Was Upregulated in BL and DLBCL Tissues and Was Associated With Poor Prognosis

**FIGURE 2:**
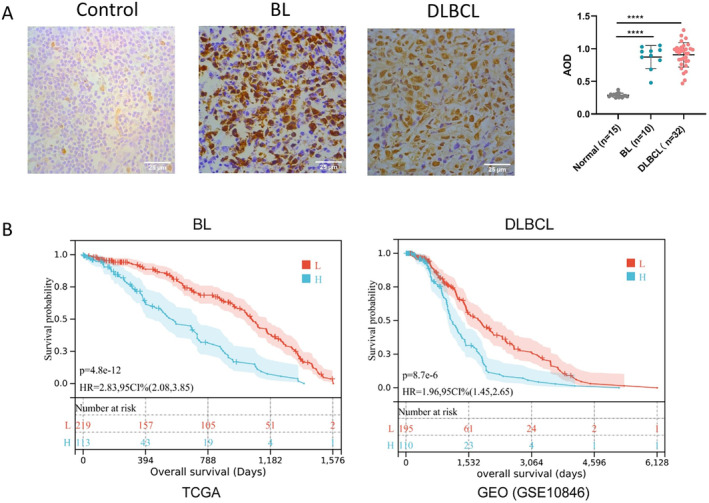
The high protein levels of CKS2 were assessed in Burkitt's lymphoma (BL) and diffuse large B‐cell lymphoma (DLBCL) tissues, and high *CKS2* expression predicted poor prognosis in BL and DLBCL patients. (A) The protein levels of CKS2 in BL and DLBCL tissues were determined by immunohistochemistry assay. Scale bar, 25 μm. (B) The association between *CKS2* expression levels and the overall survival (OS) of patients with BL and DLBCL were examined using the TCGA and GEO databases, respectively. “L” denotes low *CKS2* expression, and “H” denotes high *CKS2* expression. ****p < 0.0001. AOD, average optical density; HR, hazard ratio.

We conducted immunohistochemical (IHC) analysis to assess CKS2 protein level in samples from non‐tumor lymph nodes, BL, and DLBCL. Our findings revealed a prominent CKS2 protein level in both the nucleus and cytoplasm, with a predominant localization in the nucleus. Subsequently, the positively stained areas in a non‐tumor lymph node, BL, and DLBCL tissues were analyzed separately. Statistical analysis showed that the CKS2 protein level was significantly increased in BL and DLBCL tissues compared to non‐tumor lymph nodes (*p* < 0.0001, Figure 2A).

To investigate the correlation between the *CKS2* expression and clinical outcomes in BL and DLBCL patients, Kaplan–Meier survival analysis was conducted with data from TCGA and GSE10846. As illustrated in Figure 2B, the results indicated a significant association between *CKS2* overexpression in tumor tissues and poor overall survival (OS) in patients with BL and DLBCL (log‐rank *p* = 4.8e‐12; log‐rank *p* = 8.7e‐6).

### 3.3 PPI Network Analysis and Functional Enrichment Analysis

**FIGURE 3:**
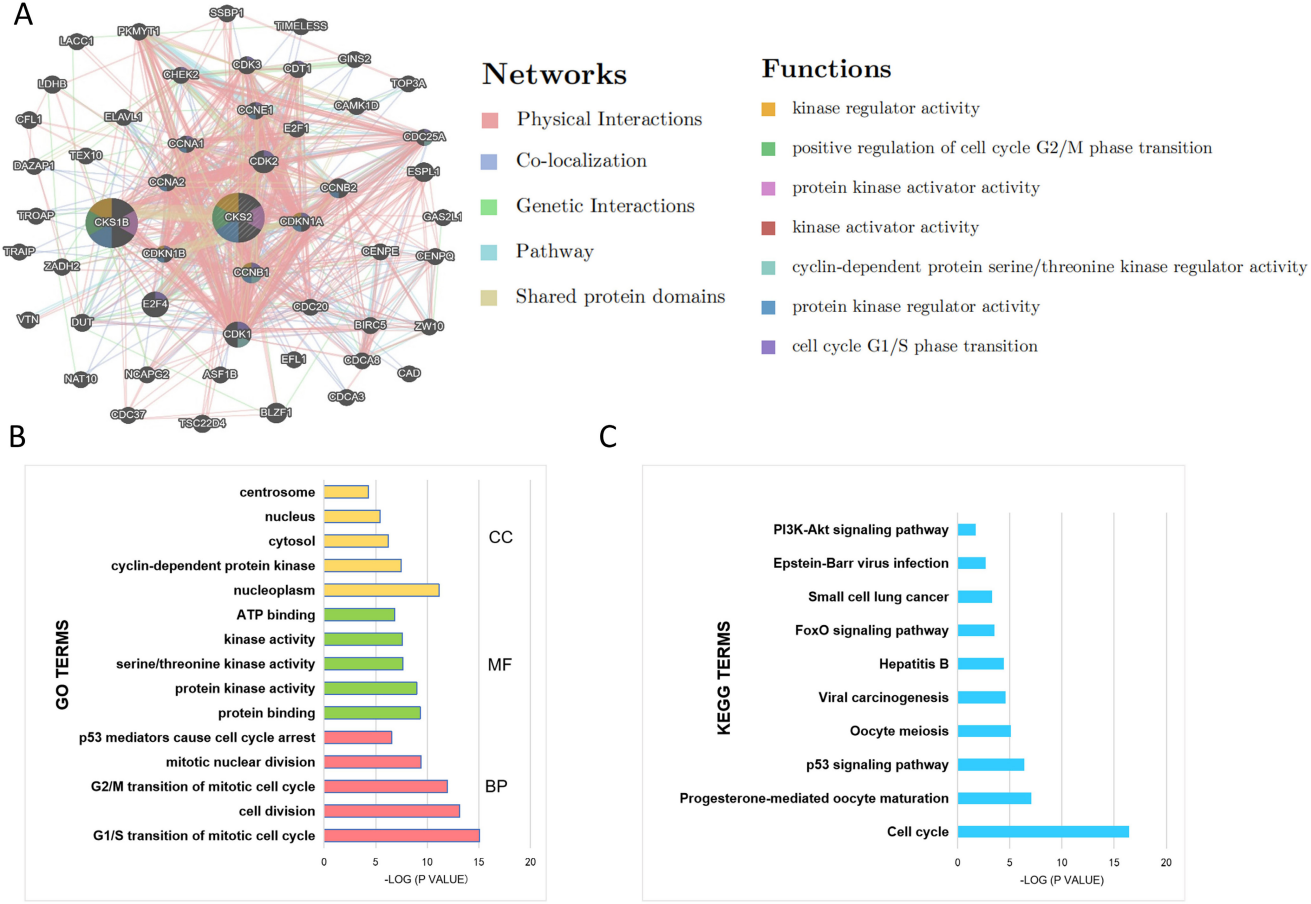
Enrichment analysis of the genes altered in the *CKS2* neighborhood. (A) Protein–protein interaction (PPI) network for *CKS2* and the first 50 adjacent genes (GeneMANIA database). Each node represented a gene, and the size of the node represented the strength of gene interaction. The color of the nodes indicated the possible function of each gene, and the color of the connecting line between the nodes represented the type of gene–gene interaction. (B, C) The top 5 GO terms (BP, MF, CC) and KEGG enrichment analysis (DAVID 6.8 database). BP, biological process; CC, cellular component; GO, Gene Ontology; KEGG, Kyoto Encyclopedia of Genes and Genomes; MF, molecular function.

To understand the possible mechanism of *CKS2* biological progression in BL and DLBCL, the GeneMANIA database was utilized to construct the PPI network for *CKS2* and its first 50 adjacent genes. As shown in Figure 3A, *CKS2* was surrounded by 50 nodes, each representing a gene, and the node size represents the strength of the gene interactions. The color of the nodes indicates the possible function of each gene, and the color of the connecting lines between the nodes indicates the type of gene–gene interaction. These nodes represented genes closely associated with *CKS2* in terms of shared protein domains, prediction, physical interactions, and co‐expression.

Then, the *CKS2* gene and the 50 adjacent genes obtained from the GeneMANIA databases were uploaded to the DAVID 6.8 database for further GO and KEGG enrichment analysis. As depicted in Figure 3B,C, the functions of these genes were associated with protein kinase regulatory activity, G1/S phase transition of the cell cycle, and p53 signaling pathway, etc.

### 3.4 ShRNA‐Mediated *CKS2* Knockdown Led to Reduced mRNA and Protein Levels in Raji and SUDHL6 Cell

**FIGURE 4:**
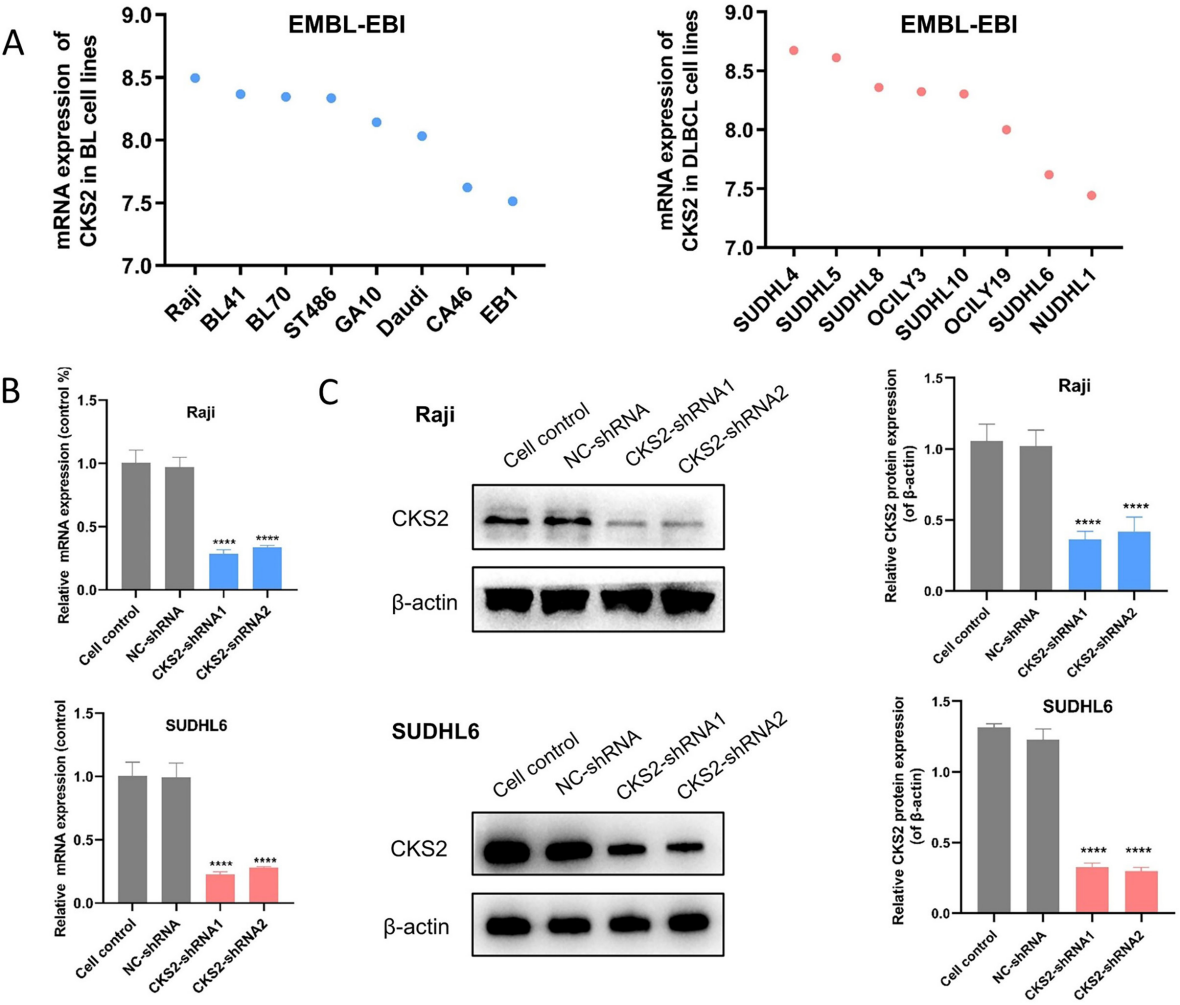
Downregulation of *CKS2* expression using shRNA in BL and DLBCL cells. (A) The mRNA expression levels of *CKS2* in 8 common BL and DLBCL cell lines (EMBL‐EBI database). (B) Raji and SUDHL6 cells were transfected with NC‐shRNA and *CKS2*‐shRNA, and the knockdown efficiency was verified by qRT‐PCR assay. (C) Raji and SUDHL6 cells were transfected with NC‐shRNA and *CKS2*‐shRNA, and the knockdown efficiency was verified by western blotting assay. ****p < 0.0001.

To ascertain whether *CKS2* expression was associated with the proliferative activity of BL and DLBCL cells, the mRNA expressions of *CKS2* were examined in the 8 common BL and DLBCL cell lines using the EMBL‐EBI database (Figure 4A). Based on the *CKS2* expression in BL and DLBCL cell lines, Raji and SUDHL6 cells were selected for further investigation. Using plasmid transfection, Raji and SUDHL6 cell lines that stably silenced *CKS2* were generated. The qRT‐PCR and western blotting were conducted to assess the ability of *CKS2*‐shRNA1 and *CKS2*‐shRNA2 to silence *CKS2* expression in vitro. As shown in Figure 4B, compared to cell control and NC‐shRNA group, *CKS2*‐shRNA1 and *CKS2*‐shRNA2 all showed significant mRNA silencing ability of CKS2 using qRT‐PCR. CKS2 protein levels were markedly downregulated by *CKS2*‐shRNA1 and *CKS2*‐shRNA2, as determined using western blotting (Figure 4C).

### 3.5 Downregulation of *CKS2* Expression Inhibited Proliferation and Induced G0/G1 Phase Arrest and Cell Apoptosis in Raji and SUDHL6 Cells

**FIGURE 5:**
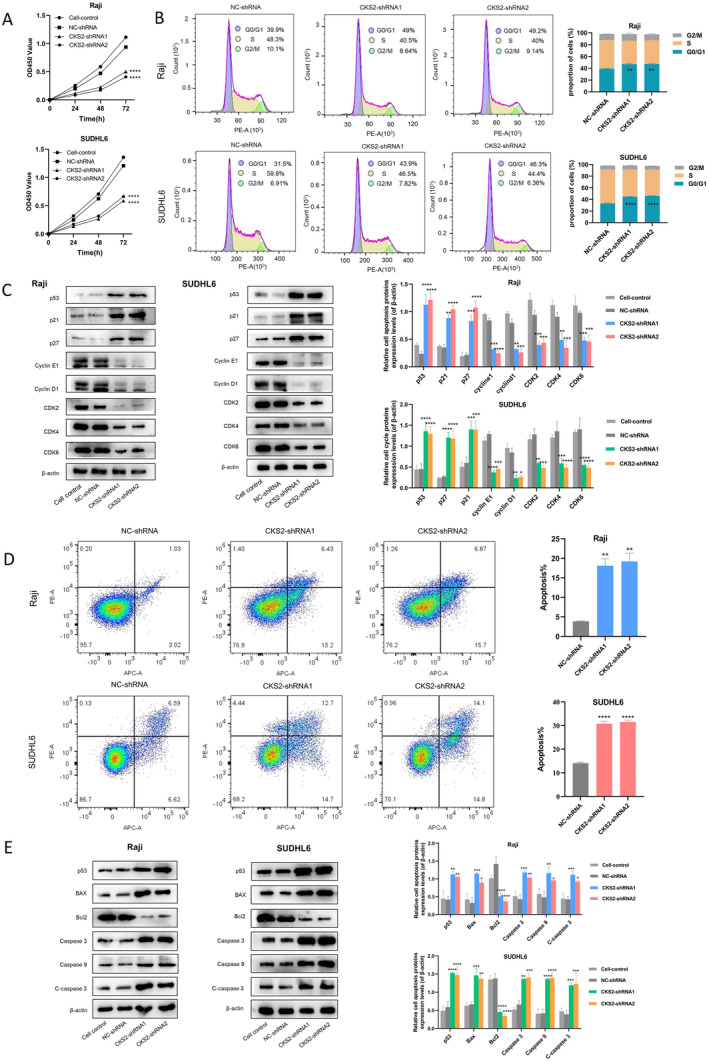
Suppression of *CKS2* inhibited proliferation and induced G0/G1 phase arrest and cell apoptosis in Raji and SUDHL6 cells. (A) Effect of *CKS2* gene silencing on the proliferation of Raji and SUDHL6 cells by Cell Counting Kit 8 assay. (B) The effects of *CKS2* knockdown on the cell‐cycle progression of Raji and SUDHL6 cells. (C) The expression levels of cell‐cycle‐related proteins were examined by western blot analysis after the knockdown of *CKS2* in Raji and SUDHL6 cells. (D) The effects of *CKS2* knockdown on promoting apoptosis of Raji and SUDHL6 cells. (E) The levels of apoptosis‐related proteins were examined by western blot analysis after knockdown of *CKS2* in Raji and SUDHL6 cells. *p < 0.05, **p < 0.01, ***p < 0.001, ****p < 0.0001.

A proliferation assay was conducted to assess whether *CKS2* depletion could impact the proliferation ability of Raji and SUDHL6 cells. As depicted in Figure 5A, the CCK‐8 assay showed that the viability of Raji and SUDHL6 cells transfected with *CKS2*‐shRNA1, *CKS2*‐shRNA2 significantly decreased from 24 to 72 h (*p* < 0.001). And there was no significant difference in cell proliferation activity between cell control and NC‐shRNA group (*p* = 0.1). This suggests that the NC‐shRNA group exerted only a minor toxic effect on Raji and SUDHL6 cells.

To elucidate the mechanism underlying the reduced proliferation in Raji and SUDHL6 cells transfected with *CKS2*‐shRNA1 and *CKS2*‐shRNA2, we assessed the cell cycle distribution using flow cytometry. The distribution of Raji cells transfected with *CKS2*‐shRNA1 and *CKS2*‐shRNA2 in the G0/G1 phase increased by 9.1% and 9.3%, respectively, compared to the NC‐shRNA group. Similarly, the distribution of SUDHL6 cells transfected with *CKS2*‐shRNA1 and *CKS2*‐shRNA2 in G0/G1 increased by 12.4% and 14.8%, respectively, compared to the NC‐shRNA group (Figure 5B). These results suggested that *CKS2*‐shRNA1, *CKS2*‐shRNA2 inhibited cell proliferation by inducing G0/G1 phase arrest. This is consistent with the results of *CKS2* functional enrichment analysis. In order to verify the influence of *CKS2*‐shRNA1 and *CKS2*‐shRNA2 on cell cycle arrest, expressions of p53, p21, p27, CDK2, CDK4, CDK6, cyclinD1, and cyclinE1 were detected by western blotting (Figure 5C). Consequently, our results supported that *CKS2*‐shRNA1 and *CKS2*‐shRNA2 could play a role in G0/G1 cell cycle arrest by upregulating protein expressions of p53/p21/p27 as well as downregulating protein expressions of CDK2/ CDK4/ CDK6/ cyclinD1/cyclinE1.

Previous studies have consistently highlighted a significant decrease in apoptosis across various lymphoma types, with abnormal or diminished apoptosis identified as a key factor contributing to the initiation and progression of lymphoma [30]. Subsequently, we aimed to explore whether the *CKS2* expression is also linked to reduced apoptosis in BL and DLBCL cells, using flow cytometry. Specifically, Raji cells transfected with *CKS2*‐shRNA1 and *CKS2*‐shRNA2 exhibited an increase in apoptosis by 17.58% and 18.52%, respectively, compared to the NC‐shRNA group (*p* < 0.01) (Figure 5D). SUDHL6 cells transfected with *CKS2*‐shRNA1 and *CKS2*‐shRNA2 exhibited an increase in apoptosis by 14.19% and 15.69%, respectively, compared to the NC‐shRNA group (*p* < 0.01) (Figure 5D). Additionally, the western blot results demonstrated a significant downregulation of Bcl2 (a well‐known anti‐apoptotic protein) expression in Raji and SUDHL6 cells transfected with *CKS2*‐shRNA1 and *CKS2*‐shRNA2, while the expression of Bax (a well‐known pro‐apoptotic protein) was markedly enhanced (Figure 5E). Furthermore, the protein levels of caspase 9, caspase 3, cleaved‐caspase 3 (C‐Caspase 3), and p53 were noticeably increased in the *CKS2*‐shRNA1 and *CKS2*‐shRNA2 groups compared to the control groups in Raji and SUDHL6 cells (Figure 5E). The aforementioned data indicate that *CKS2* knockdown increased apoptosis in both Raji and SUDHL6 cells. In summary, we found that silencing of *CKS2* expression could inhibit proliferation and induce G0/G1 phase arrest and cell apoptosis in Raji and SUDHL6 cells by activating the p53 signaling pathway. This is consistent with the results of the enrichment analysis in the figure above.

### 3.6 The Combination of *CKS2* ‐shRNA and Etoposide Exerted Synergetic Effects on Raji and SUDHL6 Cells Proliferation and Apoptosis

**FIGURE 6:**
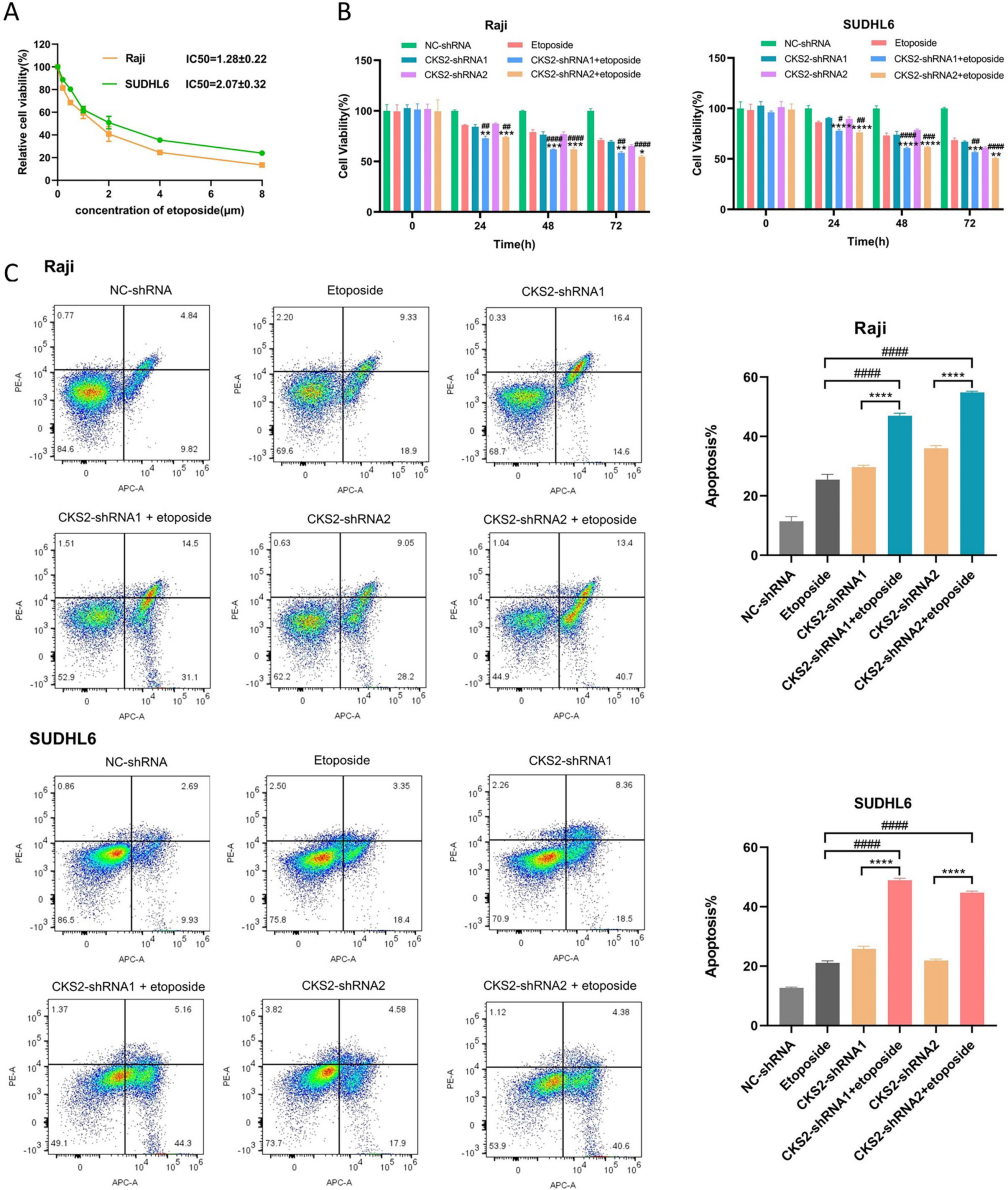
Effect of *CKS2*‐shRNA and etoposide alone and in combination on cell proliferation and apoptosis. (A) Cell viability rate at 48 h after treated with 2, 4, 6, and 8 μM etoposide. (B) Cell proliferation and (C) apoptosis of Raji and SUDHL6 cells were determined after treatment with *CKS2*‐shRNA and etoposide alone or in combination.*p < 0.05 versus *CKS2*‐shRNA, **p < 0.01 versus *CKS2*‐shRNA, ***p < 0.001 versus *CKS2*‐shRNA, ****p < 0.0001 versus *CKS2*‐shRNA; ^#^ *p* < 0.05 versus etoposide, ^##^ *p* < 0.01 versus etoposide, ^###^ *p* < 0.001 versus etoposide, ^####^ *p* < 0.0001 versus etoposide alone. Independent experiments were repeated at least in triplicate. “Etoposide” refers to treatment with NC‐shRNA + etoposide.

To assess the combined effect of *CKS2*‐shRNA and etoposide on the proliferation of Raji and SUDHL6 cells in vitro, cells were exposed to incremental concentrations of etoposide (2, 4, 6, and 8 μmol/L) for 48 h. Etoposide exhibited a dose‐dependent suppression of tumor cell growth, with IC50 values at 48 h of 1.28 ± 0.22 μM (Raji) and 2.07 ± 0.32 μM (SUDHL6) (Figure 6A). Subsequently, a CCK‐8 assay was performed on Raji and SUDHL6 cells 48 h after treatment with *CKS2*‐shRNA and etoposide alone or in combination. The results demonstrated that the cell inhibition rates induced by *CKS2*‐shRNA1 or *CKS2*‐shRNA2, either alone or in combination with etoposide, were significantly higher than those in the NC‐shRNA group (*p* < 0.05). Furthermore, the combination treatment group exhibited significantly higher inhibition rates compared to the single treatment groups (*p* < 0.05, Figure 6B).

The effects of *CKS2*‐shRNA and etoposide alone or the combination on Raji and SUDHL6 cell apoptosis were then analyzed by flow cytometry. Compared to the NC‐shRNA group, both *CKS2*‐shRNA1 and *CKS2*‐shRNA2, either alone or in combination with etoposide, can significantly induce cell apoptosis. Importantly, the combination group exhibited a significantly higher percentage of apoptotic cells compared to either drug alone (*p* < 0.0001, Figure 6C).

The mRNA expression levels of *CKS2* and *p53* in cells treated with *CKS2*‐shRNA and etoposide, either alone or in combination, were subsequently analyzed using qRT‐PCR assays. We found *CKS2* expression was decreased after etoposide treatment, and the combination group treated with *CKS2*‐shRNA and etoposide led to a lower *CKS2* expression level (Figure S1). Furthermore, the *p53* expression was increased after etoposide treatment, and the combination group treated with *CKS2*‐shRNA and etoposide led to higher *p53* expression levels (Figure S1).

## 4 Discussion

BL and DLBCL, as the two most prevalent types of B‐cell lymphomas, exhibit high aggressiveness, with their prognosis intricately linked to clinical staging, presence of lymph node metastasis, and treatment efficacy [31, 32]. Ongoing research into the pathogenesis of BL and DLBCL, alongside the development of targeted molecular therapies and exploration of effective drug combinations, is crucial for improving overall survival (OS) rates for patients. *CKS2* plays a significant role in cell cycle regulation and is implicated in tumor progression [33]. Elevated *CKS2* expression has been documented in various cancers, including MLL‐rearranged leukemia [15], bladder cancer [34], and breast cancer [35]. Studies have linked increased *CKS2* levels in malignant lymphocytes to tumor proliferation [13]. However, the underlying cellular functions of *CKS2* and the related mechanisms contributing to its carcinogenicity remain largely unexplored.

Initially, through bioinformatics analysis, we investigated *CKS2* gene expression in BL and DLBCL, analyzing its correlation with patient prognosis. Functional enrichment analysis provided insights into the molecular mechanisms involving *CKS2*. Subsequently, using shRNA‐mediated inhibition, we evaluated the impact of *CKS2* gene silencing on proliferation, cell cycle progression, and apoptosis in Raji and SUDHL6 cells. Finally, we examined the synergistic effect of *CKS2* downregulation in combination with etoposide treatment on these cells. We found that: (1) Microarray analysis revealed significantly elevated *CKS2* mRNA expression in BL and DLBCL tissues compared to normal lymph nodes or lymphocytes. Immunohistochemical staining confirmed increased CKS2 protein levels in BL and DLBCL tissues, predominantly localized in the nucleus, compared to non‐tumor lymph nodes. (2) Bioinformatics analysis indicated that upregulated *CKS2* expression correlated with poor prognosis in BL and DLBCL patients. PPI Network and functional enrichment analysis showed that *CKS2* participated in the protein kinase regulatory activity, G1/S phase transition of the cell cycle, and p53 signaling pathway, etc. (3) *CKS2* expression was successfully silenced in Raji and SUDHL6 cells using shRNAs. Silencing *CKS2* inhibited proliferation, induced G0/G1 phase arrest, and promoted apoptosis in Raji and SUDHL6 cells, potentially through activation of the p53 signaling pathway. (4) Combining *CKS2*‐shRNA with etoposide demonstrated synergistic effects on proliferation and apoptosis in Raji and SUDHL6 cells.

We initially conducted microarray data analysis and immunohistochemistry experiments, revealing significant upregulation of *CKS2* at both the transcriptome and protein levels in BL and DLBCL tissues. Kaplan–Meier survival analyses indicated that heightened *CKS2* expression predicted an unfavorable prognosis for patients. PPI network analysis and functional enrichment analysis unveiled the involvement of the *CKS2* gene in protein kinase regulatory activity, G1/S phase transition of the cell cycle, the p53 signaling pathway, and more. Remarkably, these findings align with existing literature, such as Tanaka et al.'s report [36], which associates high *CKS2* expression with biological aggressiveness and poor prognosis in gastric cancer. Studies have shown that suppressing *CKS2* expression leads to decreased cell viability, increased apoptosis, cell cycle arrest, and reduced cyclin expression in human colorectal cancer [37]. Despite previous findings suggesting the *CKS2* gene's role in promoting tumor cell proliferation in malignant lymphocytes, the precise underlying mechanism remains incompletely understood [13]. To validate these findings, we conducted functional cell experiments confirming that *CKS2* could promote proliferation in BL and DLBCL cells through regulation of the cell cycle and apoptosis. Furthermore, our investigations demonstrated that combining *CKS2* silencing with etoposide synergistically inhibited tumor proliferation and induced apoptosis in BL and DLBCL.

Since the advent of RNA interference (RNAi) in mammalian cells, short hairpin RNAs (shRNAs) have emerged as a pivotal technology for targeted gene silencing. Utilizing plasmid and viral vector systems, shRNA precursors can be transcribed and processed efficiently through the RNAi pathway, achieving robust gene knockdown [38]. In our study, *CKS2*‐shRNA1 and *CKS2*‐shRNA2 effectively silenced both mRNA and protein expression in Raji and SUDHL6 cells, chosen for in vitro tumorigenicity studies. We observed a significant reduction in cell viability following transfection with *CKS2*‐shRNA1 and *CKS2*‐shRNA2. Flow cytometry analysis revealed that *CKS2* silencing induced G0/G1 phase arrest and apoptosis in Raji and SUDHL6 cells, aligning with previous literature findings. For instance, *CKS2* inhibition has been shown to suppress proliferation, migration, invasion, and induce apoptosis in esophageal cancer cell lines [14]. Additionally, studies in human colorectal cancer have linked elevated *CKS2* expression to tumor progression and poor prognosis [37]. Mechanistically, cell cycle progression involves cyclin‐dependent kinases (CDKs) binding to cyclins. As a transcriptional target of p53, p21 inhibits Cyclin E/CDK2, thereby halting the G1/S transition upon DNA damage. Moreover, p21 enhances Cyclin D/CDK4/CDK6 kinase activity [39, 40]. *CKS2*, a downstream target of p53, is inhibited by p53 at both mRNA and protein levels, influencing cell cycle regulation [41]. *CKS2* also interacts directly with CDK1 and CDK2, further modulating cell cycle progression [42]. Our findings indicate that *CKS2* suppression induced G0/G1 phase arrest in Raji and SUDHL6 cells by downregulating CDK2, CDK4, CDK6, Cyclin E1, and Cyclin D1 while upregulating p53 and p21. In response to oncogene activation, p53 mediates apoptosis through a linear pathway involving Bax transactivation, Bax translocation from the cytosol to membranes, cytochrome c release from mitochondria, and caspase‐9 activation, followed by the activation of caspas3. p53‐mediated apoptosis can be impeded by Bcl‐2 family members regulating mitochondrial function [43]. Caspase‐3 serves as the pivotal effector in the caspase cascade governing apoptosis. Various proteases will cut the caspase‐3 zymogen, which activates caspase‐3, and the activated caspase‐3 will further cut multiple substrates, ultimately leading to cellular apoptosis [44]. In our study, low *CKS2* expression induced apoptosis in Raji and SUDHL6 cells by upregulating Bax, p53, Caspase 3, Caspase 9, and C‐Caspase 3, while downregulating BCL2 expression. Therefore, our investigation posits that *CKS2* downregulation in Raji and SUDHL6 cells may induce G0/G1 phase arrest and cell apoptosis via the p53 signaling pathway. These experimental results were consistent with the functional enrichment analysis of *CKS2*.

Combination chemotherapy holds promise for enhancing anti‐tumor effects and improving therapeutic efficacy. This approach has demonstrated notable effectiveness in the treatment of NHL. For instance, Zeng et al. reported that metronomic chemotherapy comprising prednisone, etoposide, and cyclophosphamide led to a higher objective response rate and disease control rate in patients with relapsed or refractory NHL [45]. In our study, we found that the combination of *CKS2*‐shRNA and etoposide exhibited significantly higher cell inhibition rates and induced a greater percentage of apoptotic cells compared to either treatment alone. Additionally, *CKS2*‐shRNA treatment significantly activates the p53 pathway. Interestingly, we reviewed the literature and found that etoposide could inhibit tumor proliferation in various cancers by activating the p53 pathway [46, 47]. Moreover, current research has demonstrated that the *CKS2* expression was suppressed by the tumor suppressor p53 [41]. We also found *CKS2* expression was decreased after etoposide treatment (Figure S1). Therefore, we speculate this negative feedback loop may be the reason/mechanism of the synergistic effect of *CKS2*‐shRNA and epotoside.

Our findings suggest that *CKS2*‐shRNA may serve as a sensitizer to enhance the sensitivity of tumor cells to chemotherapy drugs, providing experimental evidence for potential clinical applications in BL and DLBCL. However, it is crucial to note that further in vivo studies using relevant animal models are warranted to validate these findings. Future research efforts will focus on establishing BL and DLBCL mouse models to assess the efficacy and safety of *CKS2* inhibitors in vivo.

## 5 Conclusions

Our investigation posited that *CKS2* may play a pivotal role in the pathogenesis of BL and DLBCL, presenting compelling evidence to support the potential synergistic efficacy of combining *CKS2*‐shRNA with etoposide in therapeutic strategies targeting these malignancies.

## Author Contributions

**Chengcheng Liu:** project administration (lead), writing – review and editing (lead). **Fenling Zhou:** formal analysis (lead), investigation (lead), writing – original draft (lead). **Lu Chen:** formal analysis (lead), investigation (lead), writing – original draft (equal). **Zhen Liu:** formal analysis (equal), methodology (lead). **Yuli Cao:** data curation (lead). **Cuilan Deng:** supervision (lead). **Gexiu Liu:** project administration (lead), writing – review and editing (equal).

## Ethics Statement

The 10 BL and 32 DLBCL tissues obtained in this study were lymph node biopsy samples of patients who had not undergone radiotherapy or chemotherapy. The 15 control samples were derived from the patients' non‐tumor lymph nodes. All human tissue experiments were conducted in compliance with applicable guidelines and regulations. The Ethics Committee of Jinan University approved this study, and informed consent was obtained from all subjects and/or their legal guardians.

## Conflicts of Interest

The authors declare no conflicts of interest.

## Supporting information


Figure S1.

Figure S2.

Table S1.

## Data Availability

The data that support the findings of this study are available from the corresponding author upon reasonable request.
