# Supplementary material for: Unveiling CKS2 : A Key Player in Aggressive B‐Cell Lymphoma Progression and a Target for Synergistic Therapy
Source: Cancer Med. 2024 Nov 19;13(22):e70435. doi: 10.1002/cam4.70435 (PMC11574738; doi:10.1002/cam4.70435)
Supplement: Supplementary file 1 — Figure S1. [file CAM4-13-e70435-s001.docx]

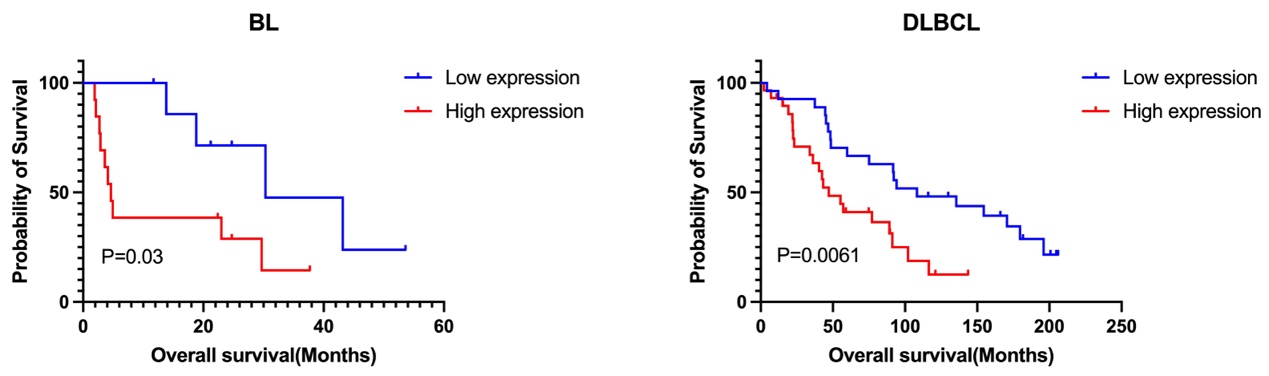


Supplementary Figure 1. The association between CKS2 expression levels and the overall survival (OS) of patients with BL and DLBCL were examined by immunohistochemistry assay, respectively. (The staining intensity of CKS2 was recorded as follows: 0 point was classified as colorless in cytoplasm or nucleus, 1 point was classified as light yellow, 2 points was classified as brownish yellow, and 3 points was classified as brown particles. Secondly, 5 representative high-magnification visual fields of positive cells were randomly selected for each specimen, and the percentage of nucleus-positive cells in each high-magnification visual field was counted, and scores were scored successively: negative cells were 0 point, <25% was 1 point, 26%~50% was 2 points, 51%~75% was 3 points, and >75% was 4 points. The positive proportion of tumor cells × staining intensity was the final result, and the optimal threshold was set as: ≥4 was classified as high CKS2 expression, <4 was classified as low CKS2 expression.)
