# Supplementary material for: Unveiling CKS2 : A Key Player in Aggressive B‐Cell Lymphoma Progression and a Target for Synergistic Therapy
Source: Cancer Med. 2024 Nov 19;13(22):e70435. doi: 10.1002/cam4.70435 (PMC11574738; doi:10.1002/cam4.70435)
Supplement: Supplementary file 2 — Figure S2. [file CAM4-13-e70435-s002.docx]

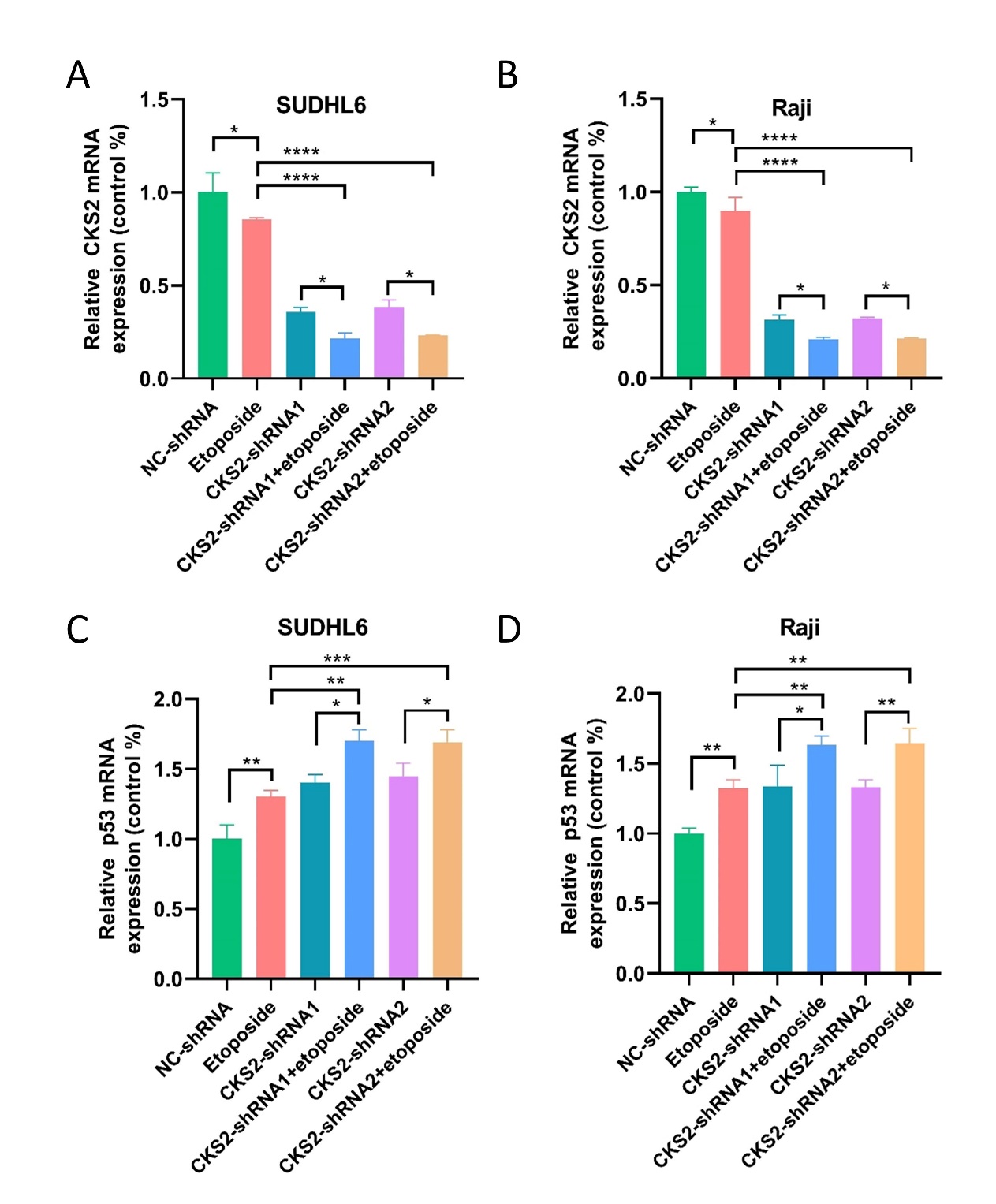


Supplementary Figure 2. The mRNA expression levels of *CKS2* and *p53* in both the single treatment group and the combination group treated with etoposide, as determined by qRT-PCR experiments. (A) The mRNA expression levels of *CKS2* in SUDHL6 cell. (B) The mRNA expression levels of *CKS2* in Raji cell. (C) The mRNA expression levels of *p53* in SUDHL6 cell. (D) The mRNA expression levels of *p53* in Raji cell. *p<0.05, **p<0.01, ***p<0.001, ****p<0.0001. Note: “Etoposide” refers to treatment with NC-shRNA + etoposide.
