# Supplementary material for: Unveiling CKS2 : A Key Player in Aggressive B‐Cell Lymphoma Progression and a Target for Synergistic Therapy
Source: Cancer Med. 2024 Nov 19;13(22):e70435. doi: 10.1002/cam4.70435 (PMC11574738; doi:10.1002/cam4.70435)
Supplement: Supplementary file 3 — Table S1. [file CAM4-13-e70435-s003.docx]

**Supplementary Table 1: Source of** **Antibodies and Reagents.**

| **Antibodies or reagents** | **Source** | **Catalog numbers** |
| --- | --- | --- |
| β-Actin | Immunoway, China | 2060 |
| p53 | Huabio, China | ET1601-13 |
| CDK2 | Huabio, China | ET1602-6 |
| CDK4 | Huabio, China | ET1612-23 |
| CDK6 | Huabio, China | ET1612-3 |
| p21 | Wanleibio, China | WL0362 |
| p27 | Wanleibio, China | WL01769 |
| Cyclin E1 | Huabio, China | ET1612-16 |
| Cyclin D1 | Huabio, China | ET1601-31 |
| Bax | Huabio, China | ET1603-34 |
| Bcl2 | Wanleibio, China | ET1603-11 |
| Caspase 3 | Huabio, China | ET1602-39 |
| C-Caspase 3 | Abcam, USA | ab2302 |
| Caspase 9 | Huabio, China | ET1701-22 |
| Goat Anti-Mouse IgG H&L (HRP) | Dia-An, China | Q1001 |
| Goat Anti-Rabbit IgG H&L (HRP) | Dia-An, China | Q1002 |
